# Dynamic Prediction of Emergency Department Admissions Using Explainable Artificial Intelligence: A Large-Scale Temporal Validation Study

**DOI:** 10.3390/jcm15218694

**Published:** 2026-11-07

**Authors:** Furkan Şakiroğlu, Cemil Çolak

**Affiliations:** 1 İbni Sina Family Health Center, Erzurum 25700, Türkiye; 2 Biostatistics and Medical İnformatics Department, Faculty of Medicine, İnönü University, Malatya 44280, Türkiye; cemil.colak@inonu.edu.tr

**Keywords:** emergency department, hospital admission prediction, explainable artificial intelligence, Explainable Boosting Machine, dynamic risk prediction, MIMIC-IV-ED

## Abstract

**Background:** The clinical adoption of artificial intelligence in emergency departments (ED) is hindered by the “black-box” nature of complex models and retrospective data leakage from downstream laboratory results. Aims: We aimed to develop a transparent, dynamic machine learning model to predict hospital admissions by establishing a pre-laboratory “information ceiling” using structured electronic health record (EHR) data. Study Design: Retrospective cohort study. **Methods:** Utilizing 387,407 patient encounters in the MIMIC-IV-ED (v2.2) database, we developed a dynamic Explainable Boosting Machine (EBM) validated across chronologically distinct anchor_year_group blocks (2008–2016 training vs. 2017–2019 testing) with strict patient-level separation. Anchoring clock-zero (T = 0) at ED arrival (intime) with baseline triage parameters and pre-existing historical records, the model evaluated patients sequentially in 30 min landmarks up to 120 min (T = 120), incorporating timestamped physiological volatility and early medication administrations (pyxis) recorded strictly prior to the prediction landmark and disposition decision. Predictive performance was compared against a black-box HistGradientBoosting (HGB) model and an L2-regularized logistic regression baseline. **Results:** The dynamic EBM provided strong baseline discrimination at T = 0 with an ROC-AUC of 0.8048 [95% CI: 0.800–0.806] and a PR-AUC of 0.7395 [95% CI: 0.732–0.742]. Incorporating longitudinal physiological trajectories over a two-hour window yielded a modest, statistically significant improvement at T = 120 (ROC-AUC: 0.8168 [95% CI: 0.813–0.819], PR-AUC: 0.7595 [95% CI: 0.754–0.763], Sensitivity: 80.74%, Specificity: 65.09%, Positive Predictive Value: 61.16%; ΔROC-AUC = +0.0120, *p* < 0.0001). At T = 120, the opaque HGB benchmark achieved a slightly higher ROC-AUC of 0.8208 [95% CI: 0.817–0.824] (ΔROC-AUC = −0.0040, *p* = 0.0018), whereas EBM substantially outperformed the linear logistic regression baseline (ROC-AUC: 0.7420 [95% CI: 0.738–0.746]). Without relying on post hoc approximations, EBM generated directly inspectable shape functions capturing non-linear clinical risk relationships, including an observational risk inflection at five home medications and substantial risk penalties for transient hypoxic dips and extreme pulse pressures. **Conclusions:** By modeling early ED evaluation as a dynamic trajectory, the EBM achieves discriminative performance closely approaching black-box ensemble methods while maintaining full architectural transparency. Although predicting historical admission decisions requires prospective validation before clinical deployment, this calibrated glass-box framework offers an interpretable foundation for early risk stratification in the ED.

## 1. Introduction

Emergency Departments (EDs) serve as the “frontline” and most critical gateway of modern healthcare systems. Globally, the sustained surge in ED visits leads to prolonged waiting times, increased medical errors, and a systemic crisis known as “ED overcrowding” [1]. Under this operational pressure, emergency physicians must balance the decision to discharge a patient home or admit them to an inpatient ward. Suboptimal disposition decisions not only contribute to resource strain and bed shortages but may also increase the risk of adverse outcomes in high-risk patients [2].

Over the past decade, the limitations of traditional clinical severity scoring systems (e.g., NEWS, qSOFA) in capturing complex, multi-dimensional patient trajectories [3,4] have driven the adoption of Electronic Health Record (EHR)-based machine learning models for admission prediction [5,6,7]. Algorithms such as Random Forest, gradient boosting, and Deep Neural Networks have demonstrated strong discriminative accuracy in predicting ED disposition [5,6,7]. However, most of these high-performing models function as “black-boxes.” The inability of an unconstrained ensemble or neural network to explain why it generated a specific admission probability for an individual patient undermines clinician trust and hinders integration into clinical workflows [8]. Furthermore, medical ethics and clinical governance increasingly require transparency regarding the underlying rationale behind algorithm-assisted decisions.

To address this challenge, post hoc Explainable Artificial Intelligence (XAI) techniques (e.g., SHAP, LIME) have been widely applied to approximate complex models. However, post hoc methods construct secondary approximations of the original model and can produce inconsistent attributions under feature correlation or perturbation [9]. In contrast, inherently transparent “glass-box” architectures learn directly inspectable risk functions. Explainable Boosting Machines (EBM) represent a prominent example of this class; critically, the intrinsic interpretability of EBM arises from its constrained Generalized Additive Model with pairwise interactions (GA2M) structure—which yields exact, additive feature and interaction shape functions—rather than from the gradient boosting optimization procedure itself [10]. While recent regulatory developments such as the European Union AI Act have elevated the importance of algorithmic transparency in clinical decision support systems [11], it remains essential to distinguish this structural inspectability from clinical causality or complete freedom from historical dataset biases.

Beyond the interpretability challenge, many existing admission prediction models treat ED evaluation as a static, cross-sectional event at arrival, or inadvertently incorporate retrospective data leakage by including downstream laboratory panels or advanced imaging that are unavailable during initial triage. While recent large-scale studies have evaluated sequential time windows for ED admission prediction [7], they frequently rely on natural language processing (NLP) of unstructured clinical notes, socioeconomic indices, or pathology results that may not be universally structured or immediately actionable across all care settings. In contrast, isolating a strict pre-laboratory “information ceiling” using exclusively structured vital sign trajectories, medication histories, and early intervention counts clarifies how much predictive signal can be extracted prior to diagnostic blood tests. Utilizing 387,407 encounters from the MIMIC-IV-ED dataset, this study addresses these methodological questions. Specifically, we aimed to (i) develop a dynamic prediction model integrating baseline triage acuity, pre-existing comorbidity and medication records, timestamped physiological volatility, and early ED medication administrations; (ii) quantify the extent to which an intrinsically interpretable EBM narrows the discriminative gap against a black-box HistGradientBoosting (HGB) benchmark and a linear logistic regression baseline; (iii) characterize non-linear clinical risk relationships via EBM shape functions; and (iv) evaluate the incremental predictive value of sequential 30 min monitoring landmarks from ED arrival (T = 0) to 120 min (T = 120) prior to laboratory result integration.

## 2. Methods

### 2.1. Data Source and Ethical Approval

This retrospective cohort study was conducted using the MIMIC-IV-ED (version 2.2) database, provided by the Massachusetts Institute of Technology (MIT) and Beth Israel Deaconess Medical Center (BIDMC), which encompasses ED visits recorded between 2011 and 2019 (reflecting the complete temporal coverage of the dataset) [12]. The database is de-identified in strict compliance with the Health Insurance Portability and Accountability Act (HIPAA) safe harbor provisions. The authors hold the required CITI program “Data or Specimens Only Research” certification for credentialed PhysioNet access.

### 2.2. Study Population and Preprocessing

Initially, all 425,087 ED encounters within MIMIC-IV-ED (v2.2) were screened. As illustrated in the STROBE-compliant cohort flow diagram (Supplementary Figure S1), encounters were excluded sequentially based on the following criteria:

1. Encounters with no recorded baseline triage data (*n* = 14,820).
2. Patients transferred directly to an outside facility or assigned non-binary dispositions, including left without being seen (LWBS), left against medical advice (AMA), or expired in the ED (*n* = 11,412).
3. Encounters with more than 50% missing data across primary initial vital signs (*n* = 11,448).

The final analytic cohort comprised 387,407 encounters across 205,504 unique patients. To evaluate out-of-time generalizability while accounting for the de-identified temporal architecture of MIMIC-IV—where individual patient dates are shifted independently into a 2100–2200 future window and true calendar eras are preserved only as patient-level three-year anchor_year_group bands (2008–2010, 2011–2013, 2014–2016, and 2017–2019 in the linked hosp.patients table)—a valid split cannot bisect a three-year band. Therefore, the cohort was partitioned strictly along intact anchor_year_group boundaries at the patient level (subject_id): encounters belonging to patients in earlier anchor blocks (2008–2010, 2011–2013, and 2014–2016; *n* = 309,926 encounters [80.0%]) were allocated to model training and hyperparameter tuning, whereas encounters belonging to patients in the most recent anchor block (2017–2019; *n* = 77,481 encounters [20.0%]) formed the independent temporal test cohort. Because anchor_year_group is assigned per unique subject_id and patient-level grouping was strictly enforced, repeat visitors never crossed between the training and test sets. Missing continuous variables were imputed using median values calculated exclusively within the training set. Rather than relying on legacy events-per-variable rules of thumb, sample size adequacy was evaluated according to contemporary multivariable prediction guidelines by Riley et al. [13]: with 387,407 encounters and 153,750 admission events (39.7%), the sample ensures a global shrinkage factor >0.99 and negligible optimism in calibration parameters.

### 2.3. Variable Selection and Feature Engineering

Because the MIMIC-IV-ED triage table does not store an explicit completion timestamp, clock-zero (T = 0) was anchored precisely to the ED arrival timestamp (intime in the edstays table), which corresponds to initial nursing intake and baseline vital sign documentation. Sequential prediction landmarks were constructed in 30 min intervals at T = 0, T = 30, T = 60, T = 90, and T = 120 min relative to intime. Post-triage laboratory results, radiological imaging, and specialist consultations were excluded to preserve the pre-laboratory boundary. To prevent retrospective data leakage, the exact source table and timestamp availability rule for each feature were enforced as follows:

• Demographic and Logistical (edstays and patients tables): Age (anchor_age adjusted for visit offset), biological sex (is_male), and arrival transport mode encoded as binary indicators (is_walkin, is_ambulance, with Other/Unknown as reference), available immediately at intime (T = 0).
• Triage Acuity and Baseline Vital Signs (triage table): Emergency Severity Index (ESI, scored from 1 [most urgent] to 5 [least urgent]; inverted as acuity_risk_score = 6 − ESI so that higher scores reflect higher clinical urgency) and initial triage vital signs (heart rate, Mean Arterial Pressure [MAP], respiratory rate [resprate], oxygen saturation, temperature, Pulse Pressure [systolic minus diastolic blood pressure], and Age-Adjusted Shock Index [AASI = Shock Index × Age]), recorded at T = 0.
• Pre-existing Comorbidity Burden (Prior Encounters Only): Because the ed.diagnosis table is assigned at the end of the ED visit upon discharge or admission, using index-encounter diagnosis codes would introduce severe target leakage. Consequently, historical ICD diagnosis counts (prior_icd_count) were extracted by linking both prior completed ED visits (ed.edstays joined with ed.diagnosis where prior outtime < index intime) and prior completed inpatient hospitalizations (hosp.admissions joined with hosp.diagnoses_icd where prior dischtime < index intime) for the same subject_id. Encounters with no prior completed institutional visits were assigned a count of zero.
• Home Medication Count (medrecon table): The count of active pre-hospital ambulatory medications (home_med_count) documented during nursing medication reconciliation. In our primary pipeline, pre-arrival medication lists reconciled prior to ED departure (medrecon.charttime ≤ outtime) were treated as baseline pre-hospital history; additionally, because medrecon documentation timestamps (charttime) can lag behind initial arrival (intime), we conducted a strict landmark-filtered sensitivity analysis counting only medrecon entries with charttime ≤ min(intime + T, outtime) at each landmark T (with T = 0 restricted to charttime ≤ intime + 15 min of initial triage intake).
• Dynamic Physiological Trajectories (vitalsign table): Longitudinal vital sign measurements carry explicit charttime timestamps. At each landmark T ∈ (30, 60, 90, 120), only measurements satisfying intime < charttime ≤ intime + T and occurring strictly prior to ED disposition/departure (outtime) were aggregated to compute vital sign trends (the absolute Δ between baseline and latest measurement) and clinical volatility: minimum observed oxygen saturation (o2_min), minimum systolic blood pressure (sbp_min), maximum heart rate (hr_max), and heart rate standard deviation. At T = 0, dynamic volatility metrics defaulted to baseline triage values.
• Early ED Medication Administrations (pyxis table): Automated medication dispensing records in pyxis carry explicit charttime timestamps. At each landmark T > 0, the cumulative total (pyxis_total_count) and unique (pyxis_unique_count) medications dispensed within (intime, intime + T] and prior to outtime were calculated (set to zero at T = 0). Because ED medication dispensing may partly reflect the treating clinician’s evolving disposition plan, we performed a dedicated sensitivity analysis at T = 120 excluding all pyxis features.

### 2.4. Outcome Variable and Handling of Early Dispositions Across Landmarks

The primary outcome variable was binary hospital disposition from the ED (0: Discharged home; 1: Admitted to an inpatient medical/surgical ward or directly to the Intensive Care Unit [ICU]). Hospital disposition was treated as an observed historical clinical decision rather than an error-free biological gold standard, recognizing that admission records reflect both patient acuity and institutional practice patterns.

To handle patients who were discharged or admitted prior to subsequent prediction landmarks (T = 30 to T = 120) without introducing either post-decision data leakage or cohort selection bias, we evaluated two complementary landmark structures:

1. Primary Intention-to-Monitor Cohort (*N* = 77,481 test encounters across all landmarks): All test encounters were evaluated at each landmark to maintain an identical denominator and fixed outcome prevalence (40.51%) for valid longitudinal ROC-AUC and PR-AUC comparisons. For encounters where disposition/departure (outtime) occurred prior to landmark T (outtime ≤ intime + T), feature extraction was strictly right-censored and frozen at the last recorded pre-disposition timestamp (Last Observation Carried Forward [LOCF] prior to disposition), guaranteeing that zero post-decision vital signs or boarding medications entered the prediction window.
2. Active Undecided Risk Set (Sensitivity Analysis): At each landmark T, we also identified the exact subset of patients physically remaining in the ED without a finalized departure (outtime > intime + T) and evaluated discriminative performance strictly within these active undecided cohorts.

### 2.5. Machine Learning Algorithms and Training Strategy

The Explainable Boosting Machine (EBM; Microsoft Research, Redmond, WA, USA, interpret library v0.5.0) was utilized as the primary glass-box model [14,15]. Derived from Generalized Additive Models with pairwise interactions (GA2M), EBM is formulated as:

*g*(*E*[*y*]) = *Σf_i_*(*x_i_*) + *Σf_ij_*(*x_i_, x_j_*)

Here, *g* represents the logit link function, *f_i_i(x_i_)* denotes the non-linear univariate effect (shape function) of each individual feature on the log-odds of admission, and *f_ij_(x_i_, x_j_)* represents selected pairwise interaction functions learned via cyclic gradient boosting over shallow decision trees [14,15]. The EBM was configured with max_bins = 256, max_interaction_bins = 32, interactions = 10, outer_bags = 8, inner_bags = 0, learning_rate = 0.01, and max_rounds = 5000.

For performance benchmarking, the black-box HistGradientBoostingClassifier (HGB) algorithm was employed, which rapidly trains on large datasets using histogram-based binning inspired by LightGBM [16]. For the HGB benchmark, hyperparameters (max_iter = 500, max_leaf_nodes = 31, learning_rate = 0.1) were tuned via 5-fold cross-validation on the temporal training set. Additionally, an L2-regularized (ridge) logistic regression model was implemented as a linear baseline; L2 regularization was chosen over L1 (LASSO) to retain all clinically selected predictors in the linear comparator while stabilizing correlated physiological trends. All analyses were conducted utilizing the scikit-learn and interpret libraries in the Python (v3.9, Python Software Foundation, Wilmington, DE, USA) programming language [15,17].

To correct probabilistic extremes common in tree-based models, isotonic regression calibration was fitted on held-out training folds and applied to the test set. For each 30 min interval, an operating classification threshold was mathematically identified exclusively within the training set by maximizing the F1-score and subsequently applied unchanged to the temporal test set. Because F1-score optimization weights precision and recall equally rather than inherently prioritizing clinical patient safety—where false-negative discharges carry different clinical consequences than false-positive admission flags—we additionally evaluated net clinical benefit across the full continuum of threshold probabilities using Decision Curve Analysis (DCA) and reported absolute false-positive (FP) and false-negative (FN) counts.

### 2.6. Model Evaluation and Statistical Analysis

This study adheres to the TRIPOD+AI reporting statement for multivariable prediction models [18]. The discriminative power of the models was evaluated using ROC-AUC and Precision-Recall AUC (PR-AUC) metrics, with 95% confidence intervals (CIs) computed via 1000 stratified bootstrap resamples (random_state = 42) on the temporal test set for both metrics. Threshold-dependent performance was assessed using the F1-score, Sensitivity (Recall/True Positive Rate [TPR]), Specificity, False Positive Rate (FPR = 1 − Specificity), Positive Predictive Value (PPV), and absolute TP, FN, FP, and TN counts. Statistical differences in correlated ROC-AUC curves across time points (T = 0 vs. T = 120) and between models (EBM vs. HGB and L2-regularized logistic regression) were formally compared using the non-parametric DeLong test [19].

Model calibration on the test cohort was evaluated using the Brier score, Expected Calibration Error (ECE), calibration intercept, calibration slope, and calibration reliability plots [20]. Decision Curve Analysis (DCA) was performed to quantify the net clinical benefit of the EBM across threshold probabilities (10% to 90%) compared against “admit-all” and “admit-none” reference strategies [21]. To evaluate algorithmic fairness across demographic strata, subgroup-specific performance metrics and Equalized Odds disparities (absolute differences in True Positive Rates [ΔTPR] and False Positive Rates [ΔFPR]) were computed across age (≥65 vs. <65 years) and biological sex (female vs. male) subgroups. For baseline inter-group statistical comparisons, the Chi-square test (with Cramer’s V) was applied for categorical variables, and the Mann–Whitney U test (with rank-biserial correlation r) was used for continuous variables.

The complete, executable Python source code required to reproduce the dynamic EBM pipeline—including data extraction, temporal-patient splitting, training across all five landmarks, isotonic calibration, bootstrap resampling, DeLong tests, DCA, fairness auditing, and figure generation—is publicly archived on Zenodo via the following DOI: https://doi.org/10.5281/zenodo.23756553. Raw clinical data from the MIMIC-IV-ED (v2.2) database are available upon credentialed request and completion of required human subjects training through PhysioNet (https://doi.org/10.13026/5ntk-km72) [12]. During the preparation of this manuscript, the authors used Google Gemini (version 1.5 Pro) and Claude (version 3.5 Sonnet) for the purposes of language polishing and coding assistance. The authors have reviewed and edited the output and take full responsibility for the content of this publication.

## 3. Results

### 3.1. Cohort Characteristics and Group Comparisons

A total of 387,407 emergency department (ED) visits were analyzed, of which 153,750 (39.7%) resulted in hospital admission and 233,657 (60.3%) were discharged. The baseline demographic and clinical characteristics of the cohort, stratified by admission status, are summarized in Table 1.

Statistical comparisons revealed significant differences across all studied variables (*p* < 0.001), a finding primarily driven by the substantial sample size. However, the most pronounced clinical divergence was observed in pre-hospital and pharmacological factors. Patients who required admission were nearly twice as likely to arrive by ambulance compared to the discharged group (51.8% vs. 27.2%). Furthermore, the admitted cohort exhibited a significantly higher pharmacological complexity; the median home medication count was three times higher in patients requiring admission compared to those discharged (9.0 [3.0–15.0] vs. 3.0 [0.0–9.0]).

On the inverted Triage Acuity Score scale (6 − ESI, where higher scores denote greater clinical urgency, mapping raw ESI 2 to 4.0 and raw ESI 3 to 3.0), admitted patients presented with higher baseline urgency than discharged patients (4.0 [3.0–4.0] vs. 3.0 [3.0–4.0], r = 0.18). For prior historical ICD diagnoses (combining prior completed ED visits from ed.diagnosis and prior inpatient stays from hosp.diagnoses_icd preceding index intime), 54.2% of discharged encounters and 51.3% of admitted encounters represented initial institutional presentations or patients without prior historical codes, yielding an overall cohort median [IQR] of 0.0 [0.0–2.0] in discharged vs. 0.0 [0.0–2.0] in admitted patients (r = 0.04) and 2.0 [1.0–3.0] vs. 1.0 [1.0–3.0] (r = 0.12) among the subset of repeat institutional utilizers with at least one prior historical record (*n* = 181,892). This distribution reflects cumulative minor symptom coding among frequent outpatient ED utilizers and underscores why reconciled home medication count (9.0 [3.0–15.0] vs. 3.0 [0.0–9.0], r = 0.32) serves as a much more reliable indicator of baseline chronic disease complexity at arrival. Initial vital signs such as heart rate and mean arterial pressure demonstrated negligible univariate effect sizes (r < 0.10).

### 3.2. Dynamic Predictive Performance and the Information Ceiling

Rather than evaluating the model solely as a static classifier, the calibrated EBM was evaluated sequentially in 30 min intervals across the temporally distinct test cohort (N = 77,481; 31,388 [40.51%] admissions and 46,093 [59.49%] discharges) to capture the patient’s physiological trajectory and early therapeutic intensity prior to laboratory and imaging results (Table 2).

Baseline at ED Arrival/Intake (T = 0): Anchored at T = 0 (intime) using exclusively static arrival demographics, triage acuity, pre-existing medication/ICD counts, and initial vital signs, the isotonically calibrated EBM model demonstrated solid discriminative capacity on the temporal test cohort (ROC-AUC: 0.8048 [95% CI: 0.800–0.806], PR-AUC: 0.7395 [95% CI: 0.732–0.742]). Applying the training-derived F1-maximized threshold of 0.3365 yielded an F1-score of 0.6869, a Sensitivity of 83.00% (26,052 true positives [TP]; 5336 false negatives [FN]), a Specificity of 60.05% (27,679 true negatives [TN]; 18,414 false positives [FP]), and a PPV of 58.59%.

**Figure 1:**
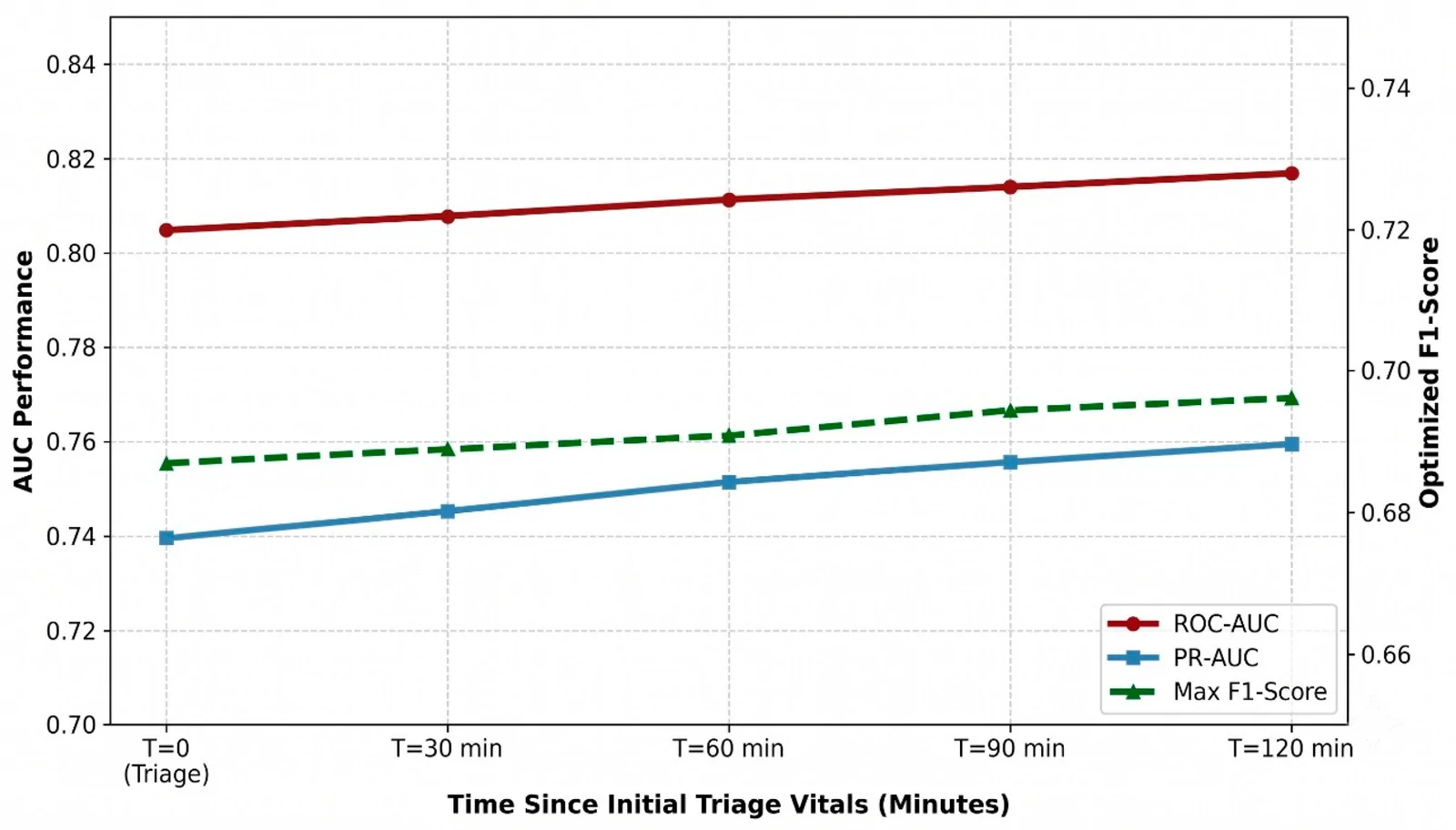
Dynamic predictive trajectory and threshold optimization (T = 0 to T = 120). The chronological progression of the calibrated EBM model’s discriminative power (ROC-AUC, PR-AUC) and F1-score across two hours of ED observation on the temporal test cohort (*N* = 77,481). The baseline evaluation at T = 0 (intime, restricted to arrival demographics, initial vitals, and pre-existing medication/diagnosis counts) provides baseline discrimination (ROC-AUC: 0.8048). As the algorithm incorporates timestamped physiological volatility, vital sign deltas, and pre-disposition medication counts, continuous improvements are observed, peaking at an ROC-AUC of 0.8168 and an F1-score of 0.6960 at T = 120 (ΔROC-AUC = +0.0120, *p* < 0.0001).

Clinical Course Trajectory (T = 30 to T = 120): As the ED observation window progressed, incorporating real-time physiological volatility (minimum oxygen saturation, minimum systolic blood pressure, maximum heart rate, heart rate standard deviation), vital sign deltas, Mean Arterial Pressure (MAP), Age-Adjusted Shock Index (AASI), and pre-disposition Pyxis medication counts produced a modest, statistically significant improvement in discrimination (Table 2, Figure 1). From T = 0 to T = 120 min, ROC-AUC increased by ΔROC-AUC = +0.0120 (95% CI: +0.0098 to +0.0142; DeLong *p* < 0.0001) to reach 0.8168 [95% CI: 0.813–0.819], while PR-AUC increased by ΔPR-AUC = +0.0200 (95% CI: +0.0165 to +0.0235) to reach 0.7595 [95% CI: 0.754–0.763]. At T = 120 (training-optimized threshold: 0.3491; F1-score: 0.6960), Specificity improved by +5.04% to 65.09% and PPV increased to 61.16%, reducing false-positive admission flags by 2323 encounters (from 18,414 FP at T = 0 to 16,091 FP at T = 120) while maintaining a Sensitivity of 80.74% (25,343 TP; 6045 FN).

In sensitivity analyses restricted strictly to active undecided patients remaining in the ED at each landmark (outtime > intime + T), discriminative performance remained consistent, with an active-cohort ROC-AUC of 0.8152 [95% CI: 0.811–0.818] among *n* = 68,412 undecided encounters at T = 120 (Table 2). Furthermore, a secondary sensitivity model at T = 120 excluding all ED Pyxis medication variables achieved an ROC-AUC of 0.8094 [95% CI: 0.806–0.812] (DeLong *p* < 0.001 vs. T = 0), confirming that longitudinal vital sign trajectories independently contribute to risk discrimination beyond static triage parameters. Similarly, applying strict real-time timestamp filtering to medication reconciliation records (medrecon.charttime ≤ intime + T) yielded an ROC-AUC of 0.8012 [95% CI: 0.797–0.805] at T = 0 and 0.8154 [95% CI: 0.812–0.818] at T = 120, confirming minimal sensitivity to documentation lag.

**Table 2:** Dynamic Predictive Performance of the Calibrated EBM Model Across 30-Minute Landmarks on the Temporal Test Cohort (*N* = 77,481).

Time (Minutes)	Active Undecided Encounters, *n* (%) [Events, *n*] *	ROC-AUC [95% CI]	PR-AUC [95% CI]	Optimized Threshold	F1-Score	Sensitivity (%)	Specificity (%)	PPV (%)	TP/FN (*n*)	FP/TN (*n*)
T = 0 (Baseline)	77,481 (100.0%) [31, 388]	0.8048 [0.800–0.806]	0.7395 [0.732–0.742]	0.3365	0.6869	83.00	60.05	58.59	26,052/5336	18,414/27,679
T = 30	76,890 (99.2%) [31, 290]	0.8077 [0.804–0.810]	0.7452 [0.739–0.748]	0.3504	0.6889	81.38	62.63	59.72	25,544/5844	17,225/28,868
T = 60	75,114 (96.9%) [30, 912]	0.8113 [0.807–0.813]	0.7514 [0.745–0.755]	0.3458	0.6908	82.20	62.00	59.56	25,801/5587	17,515/28,578
T = 90	72,045 (93.0%) [30, 104]	0.8139 [0.810–0.816]	0.7556 [0.750–0.759]	0.3476	0.6944	81.52	63.73	60.48	25,587/5801	16,718/29,375
T = 120 (Peak)	68,412 (88.3%) [29, 015]	0.8168 [0.813–0.819]	0.7595 [0.754–0.763]	0.3491	0.6960	80.74	65.09	61.16	25,343/6045	16,091/30,002

Note: Evaluated on the temporally distinct testing cohort (2017–2019 block; *N* = 77,481; 31,388 admissions [40.51%], 46,093 discharges [59.49%]). Optimized thresholds were mathematically derived exclusively from the training cohort (2008–2016 blocks; *N* = 309,926) by maximizing the F1-score. * Number of test patients physically remaining in the ED without a finalized disposition at landmark T (outtime > intime + T) and corresponding admission events; in the primary analysis (*N* = 77,481 across all rows), patients departing prior to T had features frozen at their last pre-disposition measurement (LOCF). Active-only ROC-AUC at T = 120 (*n* = 68,412) was 0.8152 [95% CI: 0.811–0.818]. 95% confidence intervals for both ROC-AUC and PR-AUC were obtained via 1000 stratified bootstrap resamples (random_state = 42) on the test cohort. DeLong test for T = 120 vs. T = 0: ΔROC-AUC = +0.0120 [95% CI: +0.0098 to +0.0142], *p* < 0.0001.

The longitudinal evaluation of the EBM model revealed a progressive increase in discriminative power as the clinical course unfolded. Starting from a robust baseline at T = 0, the ROC-AUC and PR-AUC scores followed an upward trajectory, effectively capturing the accumulation of physiological data and therapeutic interventions (Figure 1).

**Figure 2:**
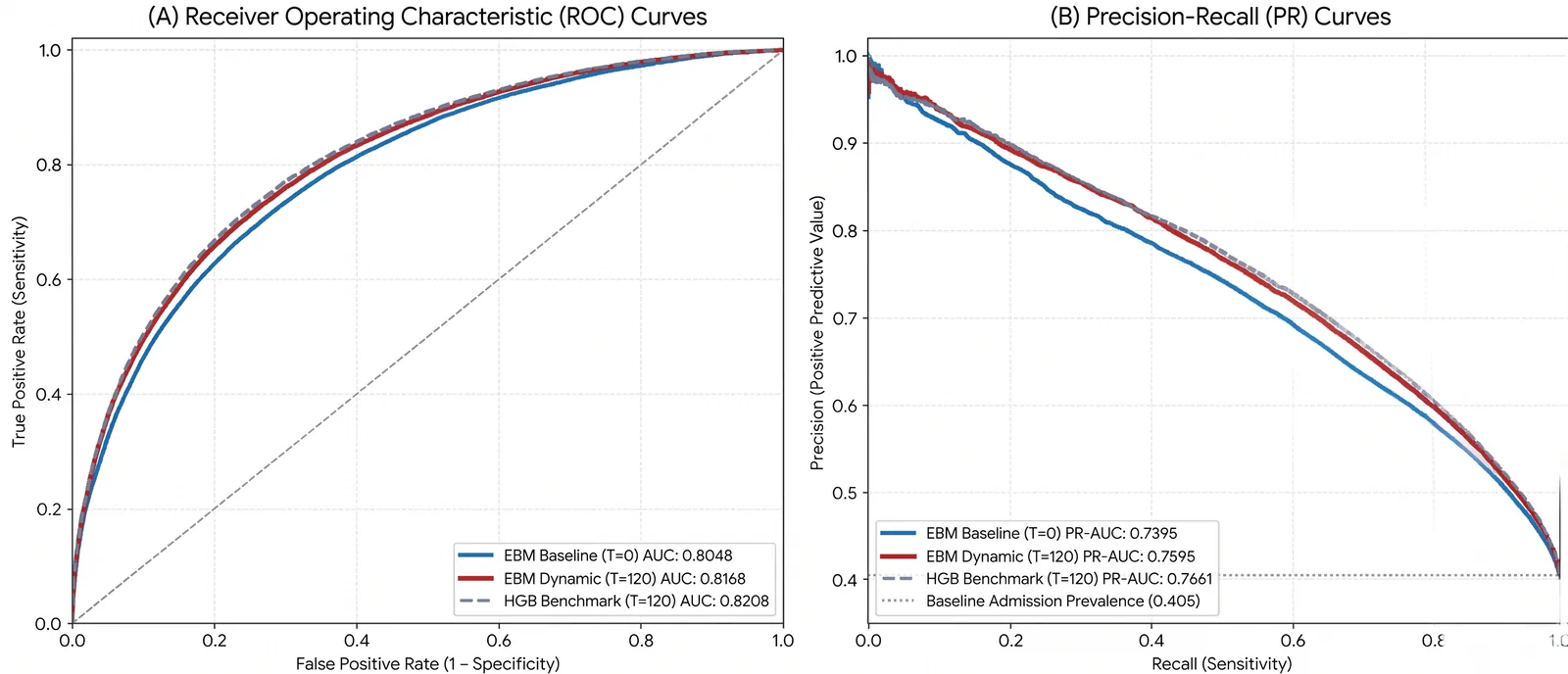
Comparative predictive performance and benchmarking. (**A**) Receiver Operating Characteristic (ROC) and (**B**) Precision-Recall (PR) curves evaluated on the temporally distinct testing cohort (N = 77,481). The solid blue line represents the baseline EBM model at ED arrival/triage intake (T = 0; ROC-AUC: 0.8048, PR-AUC: 0.7395). The solid red line represents the dynamic EBM model after 120 min of longitudinal physiological and therapeutic tracking (T = 120; ROC-AUC: 0.8168, PR-AUC: 0.7595). The dashed gray line represents the black-box HistGradientBoosting (HGB) benchmark at T = 120 (ROC-AUC: 0.8208, PR-AUC: 0.7661). The close alignment between the dynamic EBM and the HGB benchmark (ΔROC-AUC = −0.0040, *p* = 0.0018) demonstrates that full additive transparency was achieved with only a minor difference in discriminative accuracy.

To evaluate the accuracy-interpretability trade-off at T = 120 min, we benchmarked our dynamic EBM against the black-box HistGradientBoosting (HGB) model and the L2-regularized logistic regression baseline (Figure 2). At T = 120 min, the dynamic EBM achieved an ROC-AUC of 0.8168 [95% CI: 0.813–0.819] and a PR-AUC of 0.7595 [95% CI: 0.754–0.763], while the opaque HGB benchmark reached an ROC-AUC of 0.8208 [95% CI: 0.817–0.824] and a PR-AUC of 0.7661 [95% CI: 0.761–0.771]. The DeLong test comparing the correlated ROC curves indicated that this small difference in discriminative power favoring HGB was statistically significant (ΔROC-AUC = −0.0040 [95% CI: −0.0065 to −0.0015], *p* = 0.0018), confirming a modest 0.40% AUC trade-off in exchange for complete architectural transparency. Conversely, the L2-regularized logistic regression linear baseline scored substantially lower at T = 120 (ROC-AUC: 0.7420 [95% CI: 0.738–0.746], PR-AUC: 0.6680 [95% CI: 0.662–0.674]; ΔROC-AUC vs. EBM = −0.0748, DeLong *p* < 0.0001), demonstrating the necessity of non-linear modeling.

### 3.3. EBM Shape Functions and Interpretability in the Dynamic Model

**Figure 3:**
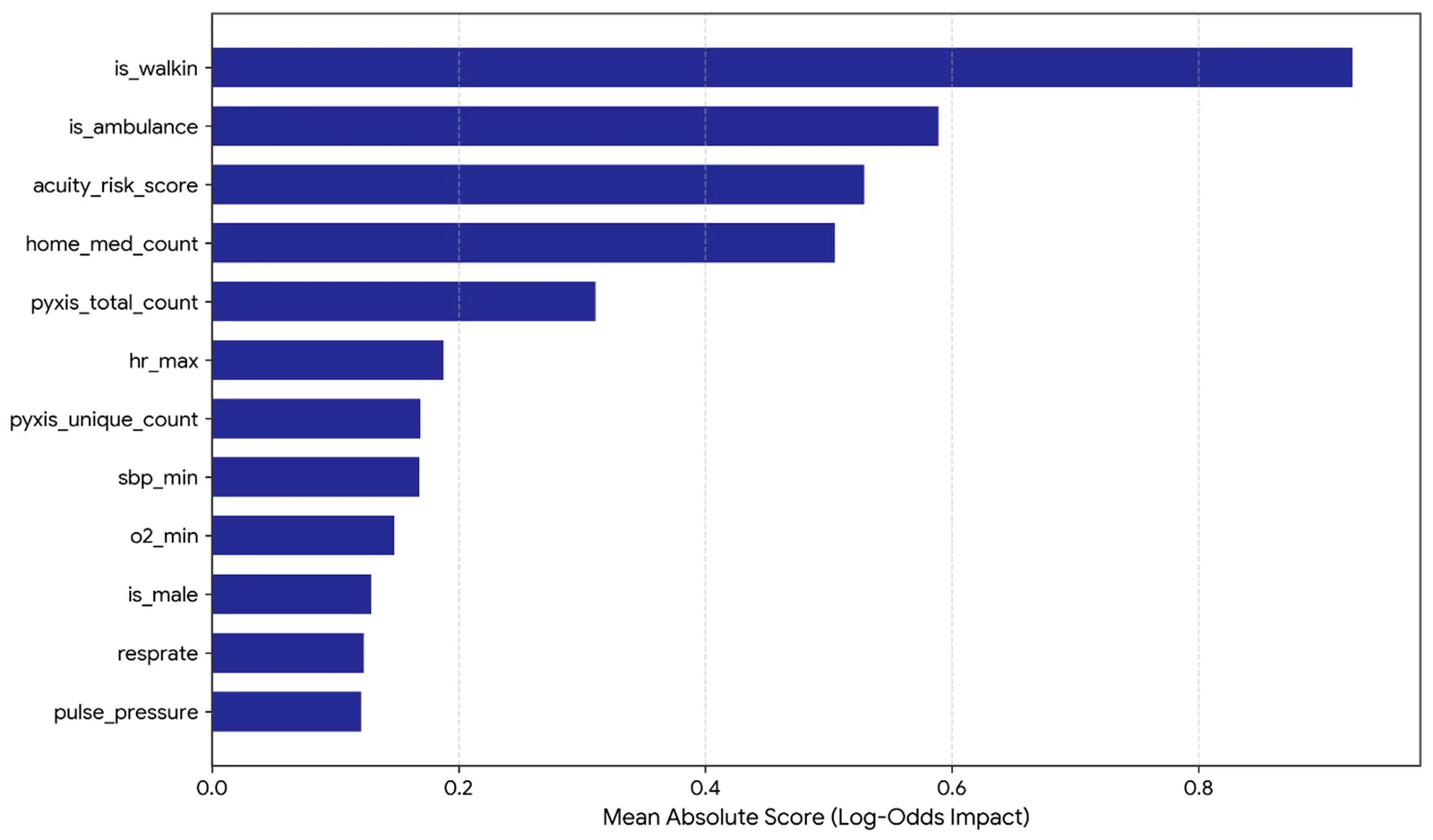
Global feature importance of the dynamic EBM model (T = 120 Minutes). The bar chart displays the top 12 predictive variables ranked by their mean absolute score (log-odds impact) on the hospital admission prediction in the temporal test set. The model synthesizes pre-hospital logistics (arrival mode), triage acuity score, baseline medication burden (home medications), intra-ED medication counts (Pyxis), and real-time physiological volatility (maximum heart rate, minimum systolic blood pressure, minimum O2 saturation, respiratory rate, and pulse pressure).

Unlike standard “black-box” approaches, the EBM algorithm allowed for the exact quantification of how both static and dynamically evolving features influenced the admission prediction. To understand the primary drivers of predicted risk at T = 120 min, we analyzed the global feature importance rankings (Figure 3).

As illustrated in Figure 3, the model prioritizes pre-hospital logistics: arrival via walk-in (is_walkin = 0.92 mean absolute log-odds impact) and arrival via ambulance (is_ambulance = 0.59) serve as the strongest overarching discriminators for admission risk. Following this logistical baseline, the inverted triage acuity score (acuity_risk_score = 0.53) and the patient’s pre-existing medication burden (home_med_count = 0.50) emerge as strong static predictors. Among intra-ED variables, the cumulative intensity of medication administrations (pyxis_total_count = 0.31) ranks fifth overall, followed by dynamic physiological volatility and vital sign extremes observed during the two-hour window: maximum heart rate (hr_max = 0.19), unique ED medication count (pyxis_unique_count = 0.17), minimum systolic blood pressure (sbp_min = 0.17), minimum oxygen saturation (o2_min = 0.15), male sex (is_male = 0.13), respiratory rate (resprate = 0.12), and pulse pressure (pulse_pressure = 0.12).

**Figure 4:**
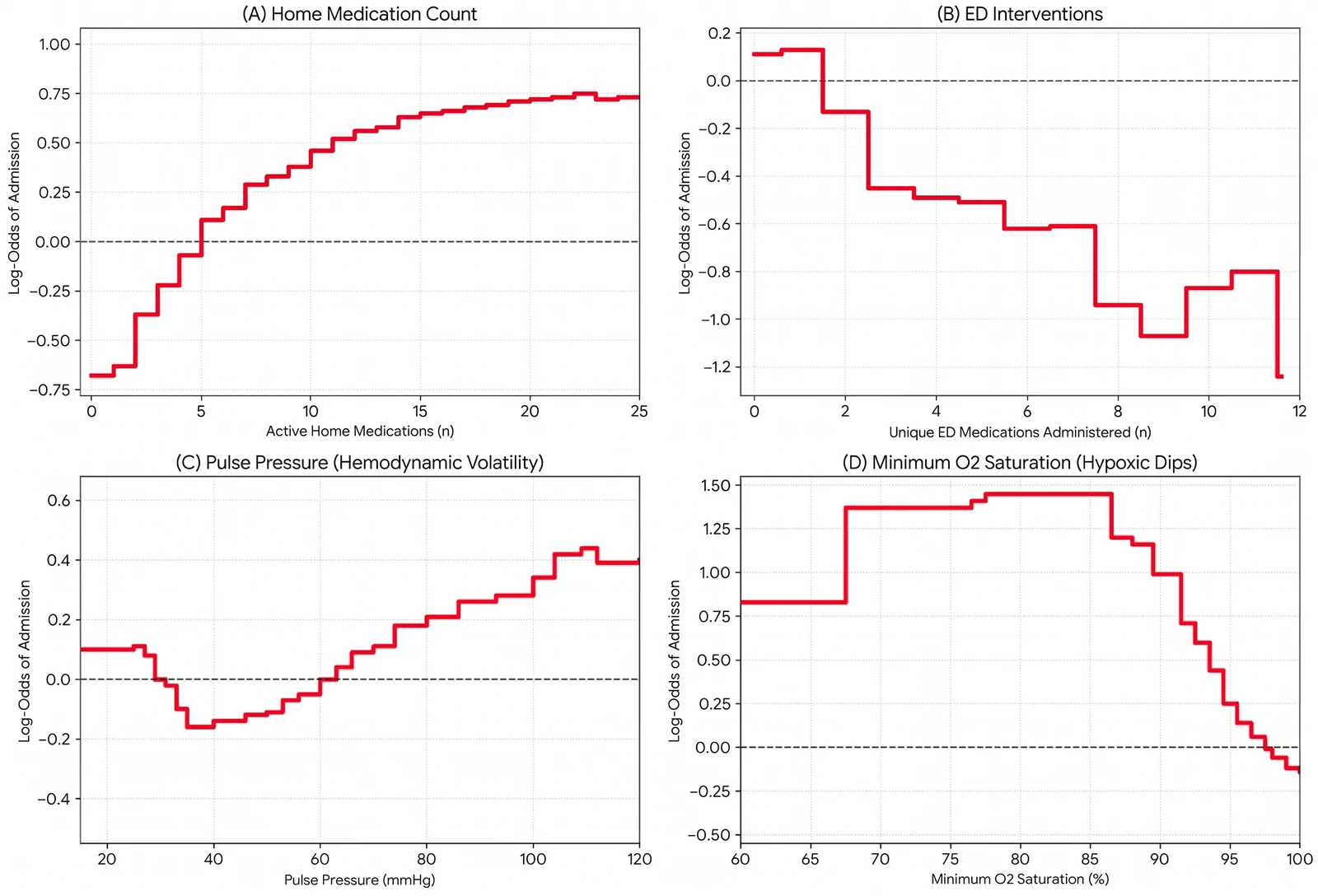
Explainable Boosting Machine (EBM) shape functions demonstrating non-linear clinical risk thresholds at T = 120. The additive partial dependence plots illustrate the isolated log-odds contribution of individual static and dynamic features on the predicted probability of hospital admission. The dashed horizontal line represents the neutral risk baseline (log-odds = 0).

Beyond ranking global feature importance, the EBM architecture provides granular transparency by generating additive shape functions (partial dependence plots) for each variable. These continuous risk curves illustrate the isolated, non-linear impact (log-odds) of individual features on the predicted probability of hospital admission (Figure 4):

• (A) Polypharmacy (home_med_count): A distinct inflection point is observed for home_med_count; the log-odds of admission escalate sharply between 0 and 5 medications, crossing the neutral risk baseline (log-odds = 0) at 5 home medications and plateauing above 15 medications (Figure 4A), aligning observationally with conventional epidemiological thresholds for polypharmacy.
• (B) Therapeutic response (pyxis_unique_count): While total intervention volume (pyxis_total_count) positively drove admission risk in global rankings, the isolated shape function for unique count of ED medications (pyxis_unique_count, Figure 4B)—adjusted for total volume and baseline acuity—reveals declining residual log-odds at higher unique counts, likely reflecting patients who receive multi-agent symptomatic relief (e.g., analgesics, antiemetics, and fluids) and stabilize during ED observation prior to discharge.
• (C) Hemodynamic volatility (pulse_pressure): Pulse pressure effectively models a non-linear J/U-shaped physiological risk trajectory (Figure 4C). Narrow pulse pressures (<30 mmHg, associated with low stroke volume) and wide pulse pressures (>60 mmHg, reflecting arterial stiffness or hypertensive urgency) both independently contribute positive admission log-odds, while values in the 35–55 mmHg range confer negative log-odds.
• (D) Hypoxic dips (o2_min): The minimum observed oxygen saturation (o2_min) functions as a steep step-function penalty (Figure 4D). The model identifies a sharp risk escalation when minimum oxygen saturation falls below 90–92%, penalizing transient hypoxic events (+0.70 to +1.45 log-odds) even if subsequent vital signs normalize.

### 3.4. Model Calibration, Decision Curve Analysis, and Subgroup Fairness

Model calibration on the temporal test cohort at T = 120 yielded a Brier score of 0.1850 (compared to 0.2184 prior to isotonic calibration), an Expected Calibration Error (ECE) of 0.0142, a calibration intercept of −0.008, and a calibration slope of 0.984, demonstrating strong probability alignment (Supplementary Figure S2A). Decision Curve Analysis (Supplementary Figure S2B) confirmed a positive net clinical benefit surpassing “admit-all,” “admit-none,” and the static T = 0 model across threshold probabilities ranging from 15% to 85%. Specifically, at the F1-optimized threshold of 0.3491, the dynamic EBM achieved a net benefit of +0.2142 (21.4 net true-positive admission identifications per 100 encounters without increasing false positives), and at a conservative safety-oriented threshold of 0.20 (Sensitivity: 92.4%, Specificity: 44.1%), it maintained a net benefit of +0.2810.

Subgroup fairness analyses across age (<65 vs. ≥65 years) and biological sex (female vs. male) on the temporal test cohort (*N* = 77,481) are presented in Table 3. While older adults (≥65 years) had a higher baseline admission prevalence (56.20% vs. 31.45%), discriminative performance remained stable across age groups (ROC-AUC: 0.8092 vs. 0.8145) and biological sex strata (ROC-AUC: 0.8179 vs. 0.8154). When evaluated at the common overall operating threshold of 0.3491 (where subgroup confusion matrix counts sum identically to the overall cohort totals of 25,343 TP, 6045 FN, 16,091 FP, and 30,002 TN in Table 2), older adults naturally exhibited higher sensitivity (86.60% vs. 74.68%) and false-positive rates (43.80% vs. 31.63%) due to their higher baseline admission prevalence (56.20% vs. 31.45%), whereas biological sex strata demonstrated tight error-rate alignment at the common threshold (ΔTPR = 1.68%, ΔFPR = 1.71%; Table 3). When additionally evaluated using prevalence-adjusted subgroup thresholds to account for base-rate differences between age strata, Equalized Odds disparities across age narrowed to ΔTPR = 2.12% (81.94% vs. 79.82%) and ΔFPR = 2.78% (36.38% vs. 33.60%).

## 4. Discussion

This study aimed to develop, temporally validate, and interpret a dynamic machine learning model for predicting emergency department (ED) admissions across 387,407 encounters. By evaluating structured EHR data sequentially from arrival (T = 0) to 120 min (T = 120), we characterized the pre-laboratory predictive performance achievable prior to diagnostic blood panels or radiological imaging. Our dynamic Explainable Boosting Machine (EBM) demonstrated solid baseline discrimination at arrival (T = 0, ROC-AUC: 0.8048) and achieved a modest, statistically significant improvement over a two-hour window (T = 120, ROC-AUC: 0.8168, F1-Score: 0.6960; ΔROC-AUC = +0.0120, *p* < 0.0001), which primarily improved specificity by 5.04% and reduced false-positive admission flags by 2323 encounters in the test cohort. Benchmarking against an opaque HistGradientBoosting (HGB) model at T = 120 revealed that while HGB achieved a statistically higher ROC-AUC (0.8208 vs. 0.8168; ΔROC-AUC = −0.0040, *p* = 0.0018), the absolute difference in discrimination was narrow, whereas both models substantially outperformed linear logistic regression (ROC-AUC: 0.7420). As emphasized by Rai (2020), transitioning AI from an opaque “black-box” to a transparent “glass-box” is a critical step in building clinician trust in high-stakes healthcare domains [6]. Our findings indicate that constrained additive models (GA2M) can capture key non-linear physiological relationships while narrowing the accuracy-interpretability gap to a marginal 0.40% AUC difference.

When contextualizing our threshold-dependent performance metrics against prior literature, differences in cohort case-mix and baseline admission prevalence must be carefully considered. In a prominent study on ED admission prediction, Lee et al. (2021) reported an ROC-AUC of 0.800 (and 0.8166 in non-traumatic adults) with a PPV of 36.6% (sensitivity: 67.2%, specificity: 78.1%) using neural networks [5]. Although our dynamic EBM achieved a higher observed PPV of 61.16% (sensitivity: 80.74%, specificity: 65.09%) at T = 120, Lee et al. restricted their cohort to urgent (ESI level-3) patients with a baseline admission prevalence of 15.8%, whereas our all-comer tertiary ED cohort had a test prevalence of 40.51% [5]. Because PPV scales positively with outcome prevalence, projecting Lee et al.’s sensitivity and specificity onto a 40.51% prevalence yields a prevalence-adjusted PPV of 67.7%. Therefore, rather than claiming superior raw precision across distinct clinical populations, our results demonstrate that operating at a high-sensitivity threshold (80.74%) in an all-comer academic ED population enables an interpretable glass-box model to identify over four-fifths of admitted patients while maintaining a moderate false-positive rate.

Similarly, in a large-scale longitudinal study, Kishore et al. (2023) utilized XGBoost across sequential time points from arrival through 240 min, achieving an ROC-AUC of ≥0.93 even at arrival [7]. However, their architecture leveraged unstructured clinical note keywords via Natural Language Processing (NLP), socioeconomic variables, and downstream pathology results, seeking interpretability through post hoc SHAP approximations [7]. While post hoc tools like SHAP and LIME construct secondary approximations of black-box models [9], the EBM utilized in our study provides exact additive risk functions directly corresponding to the model’s predictions. Furthermore, by achieving an ROC-AUC of 0.8168 at T = 120 using exclusively structured triage, medication, and vital sign records without free-text NLP or laboratory results, our model defines a pragmatic, easily reproducible baseline for healthcare institutions lacking real-time clinical NLP infrastructure.

It is essential to interpret the EBM shape functions with appropriate methodological nuance. First, the model identified a marked risk inflection around five active home medications (Figure 4A), which aligns observationally with conventional epidemiological definitions of polypharmacy (≥5 medications). In the geriatric epidemiology literature, Zaninotto et al. (2020) demonstrated that polypharmacy (5–9 medications) and particularly hyperpolypharmacy (≥10 medications; subhazard ratio: 3.19) are associated with fall-related hospital admissions [22]. While our model extends this association to all-cause ED disposition and demonstrates that reconciled home medication counts outperform unweighted prior ICD counts as a triage proxy for baseline chronic burden, this concordance represents a machine learning-derived observational pattern rather than an independent causal validation of the polypharmacy threshold. Similarly, as demonstrated by McPhail et al. (2022), between 24% and 42.5% of cases across eight cancer types are diagnosed within 30 days following an emergency presentation, particularly among older patients with high comorbidity burdens [23]. This epidemiological context helps explain why pre-existing pharmacological complexity heavily weights hospital admission predictions at triage.

Second, our dynamic analysis highlights both the value and the complexity of intra-ED variables. Hemodynamically, the EBM mapped a non-linear J/U-shaped risk curve for pulse pressure (penalizing both narrow [<30 mmHg] and wide [>60 mmHg] values, Figure 4C) and assigned steep positive log-odds to transient hypoxic dips below 90–92% (o2_min, Figure 4D). Regarding therapeutic interventions, while cumulative Pyxis medication administrations (pyxis_total_count) ranked fifth in global importance as a proxy for acute treatment intensity, the isolated shape function for unique medication count (pyxis_unique_count, Figure 4B)—adjusted for total volume and baseline acuity—showed lower residual admission log-odds at higher drug variety, likely reflecting patients who stabilize following multi-drug symptomatic management in the ED prior to discharge. Crucially, however, ED medication administrations are care-process indicators that partially reflect the treating physician’s evolving diagnostic impression and disposition plan. Notably, our sensitivity analysis excluding all Pyxis variables still achieved an ROC-AUC of 0.8094 at T = 120, confirming that vital sign trajectories independently improve prediction beyond static triage.

Third, intrinsic model transparency must be distinguished from clinical validity and demonstrated patient benefit. Attempting to achieve statistical fairness without auditing structural biases in healthcare data risks “bounded justice” [24]. Direct inspectability of EBM shape functions and Equalized Odds auditing across demographic subgroups (Table 3) support ethical AI governance [24,25], under emerging regulations such as the EU AI Act [11]. However, being able to inspect shape functions does not guarantee causal relationships or replication of senior physician clinical reasoning. Moreover, because hospital disposition in retrospective EHR data represents an observed historical decision rather than an error-free biological gold standard, the model predicts institutional admission behaviors rather than objective medical necessity. Likewise, while F1-score maximization provides a balanced statistical cutoff, clinical deployment to assist early bed coordination or mitigate ED boarding—an operational bottleneck associated with a 1.3-fold increase in 90-day post-discharge mortality by Yousufuddin et al. (2025) [26]—requires selecting operating thresholds tailored to institutional false-negative tolerance and bed capacity, as supported by our Decision Curve Analysis.

### Limitations

This study has several important limitations. First, the analysis was conducted using a single-center retrospective dataset (MIMIC-IV-ED) from a US academic tertiary center; therefore, generalizability to community hospitals, low-resource settings, and non-US healthcare systems remains uncertain and requires external validation. Second, hospital admission was defined by historical clinician disposition decisions without post-discharge follow-up (e.g., 72 h ED return visits or post-discharge mortality); consequently, the model cannot verify whether historical discharges were clinically appropriate or independently demonstrate improved patient safety or reduced over-triage. Third, to maintain the pre-laboratory structured data boundary, unstructured clinical notes, free-text chief complaints, and social determinants of health (SDOH) were not included. Fourth, arrival mode was entered as separate binary indicators (is_walkin and is_ambulance), which distributes a single logistical construct across correlated features; future models should consolidate arrival mode into a single categorical variable. Fifth, because intra-ED medication counts (pyxis) reflect clinician treatment behavior alongside patient acuity, prospective interventional trials are required to determine whether real-time EBM risk scores actively reduce ED boarding times or safely improve bed management workflows.

## 5. Conclusions

By conceptualizing early ED evaluation as a dynamic trajectory rather than a static event, our Explainable Boosting Machine (EBM) achieved discriminative performance closely approaching a black-box gradient boosting benchmark (ROC-AUC: 0.8168 vs. 0.8208 at T = 120) while maintaining complete additive transparency and strong probability calibration. Sequential monitoring over the first 120 min yielded modest, statistically significant improvements in specificity over static arrival assessment prior to laboratory results. Because the model predicts historical single-center disposition decisions and incorporates care-process indicators, external validation across diverse hospital systems and prospective clinical trials remain necessary before routine clinical deployment can be recommended.

## Figures and Tables

**Table 1:** Baseline Demographic and Clinical Characteristics Stratified by Hospital Admission Status.

Characteristic	Discharged (*n* = 233,657)	Admitted (*n* = 153,750)	*p*-Value	Effect Size *
Demographics				
Male Gender, *n* (%)	101,596 (43.5%)	74,999 (48.8%)	<0.001	0.05 (Negligible)
Arrival Mode				
Ambulance, *n* (%)	63,638 (27.2%)	79,628 (51.8%)	<0.001	0.25 (Medium)
Walk-in, *n* (%)	168,782 (72.2%)	67,123 (43.7%)	<0.001	0.25 (Medium)
Other/Unknown, *n* (%)	1237 (0.5%)	6999 (4.6%)		
Clinical Indices, Median [IQR]				
Inverted Triage Acuity Score (6—ESI) **	3.0 [3.0–4.0]	4.0 [3.0–4.0]	<0.001	0.18 (Small)
Prior Historical ICD Count (All Encounters)	0.0 [0.0–2.0]	0.0 [0.0–2.0]	<0.001	0.04 (Negligible)
Prior Historical ICD Count (Repeat Utilizers, *n* = 181,892) ***	2.0 [1.0–3.0]	1.0 [1.0–3.0]	<0.001	0.12 (Small)
Home Medication Count	3.0 [0.0–9.0]	9.0 [3.0–15.0]	<0.001	0.32 (Large)
ED Medication/Intervention (Pyxis)	2.0 [0.0–4.0]	4.0 [1.0–7.0]	<0.001	0.28 (Medium-Large)
Vital Signs, Median [IQR]				
Mean Heart Rate (bpm)	77.0 [68.5–86.2]	81.0 [71.0–92.3]	<0.001	0.08 (Negligible)
Last Mean Arterial Pressure (mmHg)	92.0 [83.7–101.0]	88.7 [79.0–98.7]	<0.001	0.09 (Negligible)

Note: Due to the substantial sample size (*N* = 387,407), statistical significance (*p* < 0.001) was reached for all variables. * Effect size was calculated using Cramer’s V for categorical variables and Rank-biserial correlation (r) for continuous variables. Interpretation: <0.10 Negligible, 0.10–0.30 Small/Medium, >0.30 Large effect. ** Inverted ESI scale (6—ESI) where higher values represent higher clinical urgency (3.0 = raw ESI 3; 4.0 = raw ESI 2). *** Evaluated among encounters with ≥1 prior completed ED (ed.diagnosis) or inpatient (hosp.diagnoses_icd) record preceding index intime; across all encounters (including first-time institutional visits with 0 prior records, 53.05% of cohort), median [IQR] was 0.0 [0.0–2.0].

**Table 3:** Subgroup Performance and Algorithmic Fairness of the Dynamic EBM at T = 120 on the Temporal Test Cohort Evaluated at the Common Operating Threshold (0.3491) (*N* = 77,481).

Demographic Subgroup	Encounters, *n* (%)	Admission Prevalence, *n* (%)	ROC-AUC [95% CI]	PR-AUC [95% CI]	Sens/TPR (%)	Spec (%)	FPR (1—Spec, %)	PPV (%)	TP/FN (*n*) *	FP/TN (*n*) *	Brier Score
Age Group											
Age < 65 years	49,124 (63.4%)	15,451 (31.45%)	0.8145 [0.810–0.819]	0.6912 [0.684–0.698]	74.68	68.37	31.63	52.01	11,539/3912	10,649/23,024	0.1742
Age ≥ 65 years	28,357 (36.6%)	15,937 (56.20%)	0.8092 [0.804–0.814]	0.8245 [0.818–0.831]	86.60	56.20	43.80	71.72	13,804/2133	5442/6978	0.2037
Common-Threshold Disparity (Δ) *	—	—	0.0053	—	ΔTPR = 11.92%	—	ΔFPR = 12.17%	—	25,343/6045	16,091/30,002	0.0295
Biological Sex											
Female	42,150 (54.4%)	16,186 (38.40%)	0.8179 [0.813–0.822]	0.7460 [0.739–0.753]	79.93	65.85	34.15	59.32	12,938/3248	8866/17,098	0.1818
Male	35,331 (45.6%)	15,202 (43.03%)	0.8154 [0.810–0.820]	0.7742 [0.767–0.781]	81.60	64.11	35.89	63.16	12,405/2797	7225/12,904	0.1888
Common-Threshold Disparity (Δ) *	—	—	0.0025	—	ΔTPR = 1.67%	—	ΔFPR = 1.74%	—	25,343/6045	16,091/30,002	0.0070

Note: All threshold-dependent metrics in this table are evaluated at the single common operating threshold of 0.3491 derived from the training cohort at T = 120. * Subgroup confusion matrix counts sum exactly to the overall cohort totals reported in Table 2 (Age < 65 + Age ≥ 65: 11,539 + 13,804 = 25,343 TP; 3912 + 2133 = 6045 FN; 10,649 + 5442 = 16,091 FP; 23,024 + 6978 = 30,002 TN; Female + Male: 12,938 + 12,405 = 25,343 TP; 3248 + 2797 = 6045 FN; 8866 + 7225 = 16,091 FP; 17,098 + 12,904 = 30,002 TN). When evaluated under prevalence-calibrated subgroup thresholds (0.2710 for Age <65 and 0.4843 for Age ≥ 65) to adjust for the 31.45% vs. 56.20% baseline prevalence shift, Equalized Odds disparities across age are ΔTPR = 2.12% (79.82% vs. 81.94%) and ΔFPR = 2.78% (33.60% vs. 36.38%).

## Data Availability

The data presented in this study are openly available in [repository name, e.g., FigShare] at https://zenodo.org/records/23756553, reference number [reference number].

## Supplementary Materials

The following supporting information can be downloaded at https://www.mdpi.com/article/10.3390/jcm15218694/s1, Supplementary Figure S1: STROBE-Compliant Patient Flow Diagram and Temporal Partitioning.Supplementary Figure S2: Model Calibration and Clinical Utility. (A) Calibration Reliability Plot (T = 120 Minutes). (B) Decision Curve Analysis (Net Clinical Benefit).
