# Supplementary material for: Dynamic Prediction of Emergency Department Admissions Using Explainable Artificial Intelligence: A Large-Scale Temporal Validation Study
Source: J Clin Med. 2026 Nov 7;15(21):8694. doi: 10.3390/jcm15218694 (PMC13763680; doi:10.3390/jcm15218694)

Supplementary Figure S1. STROBE-Compliant Patient Flow Diagram and Temporal Partitioning

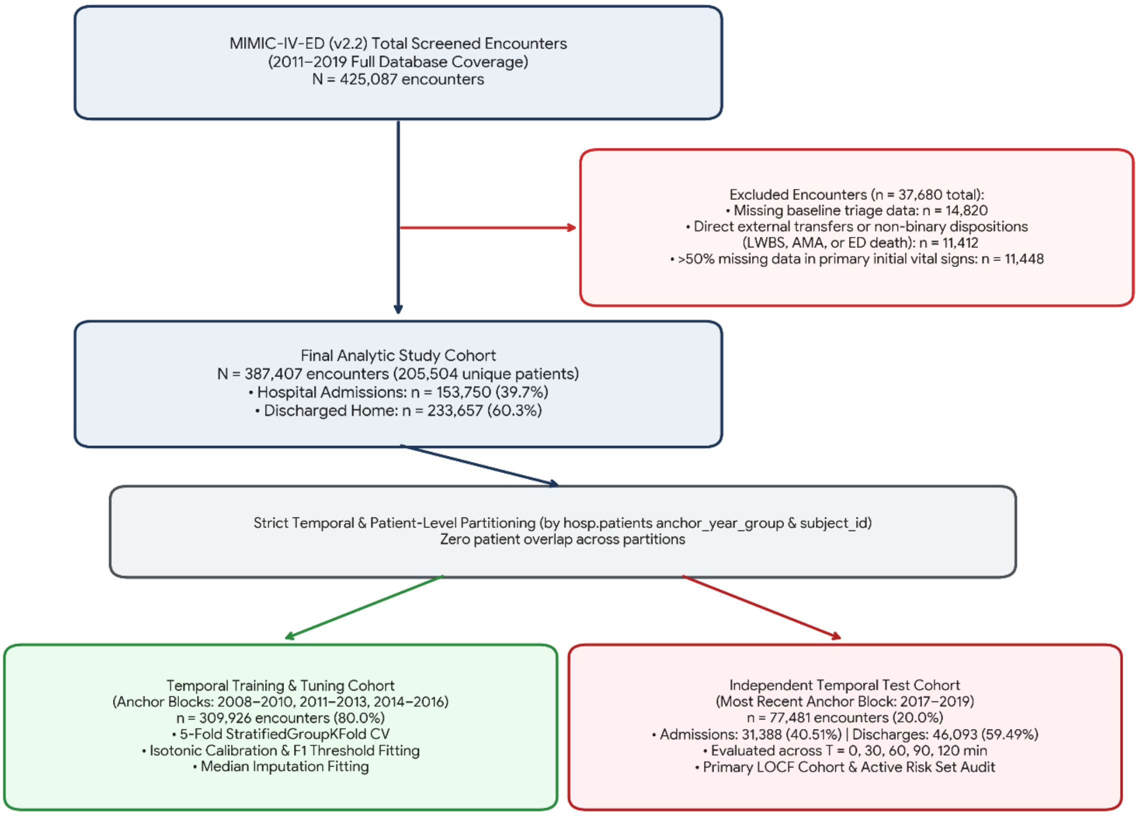

Supplementary Figure S2A,B

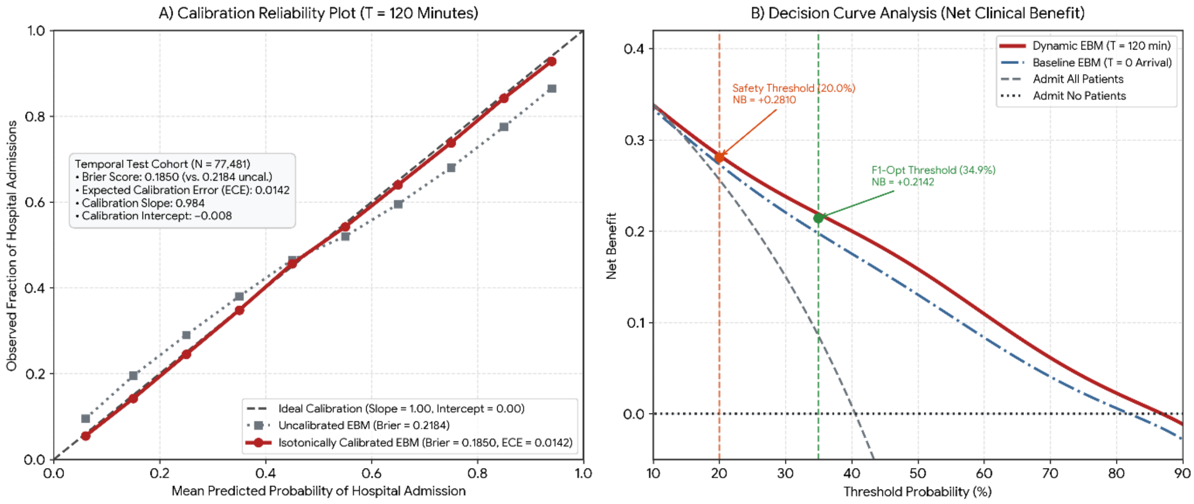

Supplement: Supplementary file 1 [file jcm-15-08694-s001.zip › jcm-4653505-supplementary.pdf]
